# *Styphnolobium japonicum* (L.) Schott Fruits Increase Stress Resistance and Exert Antioxidant Properties in *Caenorhabditis elegans* and Mouse Models

**DOI:** 10.3390/molecules24142633

**Published:** 2019-07-19

**Authors:** Sara Thabit, Heba Handoussa, Mariana Roxo, Bruna Cestari de Azevedo, Nesrine S.E. El Sayed, Michael Wink

**Affiliations:** 1 Pharmaceutical Biology Department, Faculty of Pharmacy and Biotechnology, German University in Cairo, Cairo 11835, Egypt; 2 Biology Department, Institute of Pharmacy and Molecular Biotechnology, Heidelberg University, Im Neuenheimer Feld 364, Heidelberg 69120, Germany; 3 Departmento de Biotecnologia em Plantas Medicinais, Universidade de Ribeirão Preto, 14096-900 Ribeirão Preto, Brazil; 4 Pharmacology and Toxicology department, Faculty of Pharmacy, Cairo University, Cairo 11562, Egypt

**Keywords:** *Styphnolobium japonicum*, *Caenorhabditis elegans*, oxidative stress, antioxidant, HPLC-PDA-ESI-MS/MS

## Abstract

*Styphnolobium japonicum* (L.) Schott is a popular Asian tree widely used in traditional medicine. The current study explored the potential stress resistance and antioxidant activities of its fruits. Phytochemical profiling of the hydroalcoholic fruit extract was done via high performance liquid chromatography-photodiode array-electrospray ionization-mass/mass (HPLC-PDA-ESI-MS/MS). Twenty four phenolic constituents were tentatively identified in the extract. The *Caenorhabditis elegans* (*C. elegans*) nematode model in addition to trimethyltin (TMT)-induced neurotoxicity mouse model were used for in vivo evaluation of its antioxidant properties. The ability of the extract to enhance stress resistance was manifested through increasing survival rate by 44.7% and decreasing basal reactive oxygen species (ROS) levels by 72.3% in *C. elegans*. In addition, the extract increased the levels of the stress response enzyme superoxide dismutase-3 (Sod-3) by 55.5% and decreased the expression of heat shock protein-16.2 (Hsp-16.2) in nematodes, which had been challenged by juglone, by 21%. Using a mouse model, the extract significantly decreased the expression of the oxidative stress marker malondialdehyde (MDA). Furthermore, an elevation in the levels of the antioxidant marker glutathione (GSH), SOD and heme oxygenase-1 (HO-1) enzymes were observed. Our findings imply that *Styphnolobium japonicum* has the potential to be used in future studies focusing on diseases associated with oxidative stress.

## 1. Introduction

*Styphnolobium japonicum* (SJ) (L.) Schott (formerly *Sophora japonica*), also called Chinese scholar tree and Japanese pagoda tree, is a deciduous tree that belongs to family Fabaceae. Though this tree originated in China, it is found as an ornamental plant in other areas like Eastern Asia, Europe, South Africa, and North America [1]. It is widely known for its use in traditional Chinese medicine for treating dizziness, headache, hypertension, hematemesis, intestinal hemorrhage, and hemorrhoids [1,2]. Several parts of the plant are used pharmaceutically as Sophorae fructus, Sophorae flos, and Sophorae gemmae [3]. Extracts from flower buds and the isolated rutin are used in phytotherapy to treat symptoms of capillary and venous insufficiency, including swollen legs, varicose veins, cramps, and piles [1,4]. Extracts of different plant parts have also shown astringent, antibacterial, antispasmodic, hypotensive, anticholesterolemic, and anti-inflammatory properties [2,5,6,7,8,9].

Several phytochemical studies of SJ have revealed the presence of many secondary metabolites like flavonoids, triterpenoids, and amino acids, but most of the pharmacological properties are attributed to both flavonoids and isoflavonoids [1]. It is also noteworthy that rutin represents 20% of SJ flower buds dry weight [3]. 

Several studies addressed the pharmacological activities of extracts from different parts of SJ. Fruit extracts have shown an inhibitory effect on the activity of osteoclasts, leading to prevention of bone loss. Dichloromethane extract of the fruits, rich in genistein, has demonstrated osteogenic properties via promoting differentiation of osteoblasts [10]. The estrogenic compound sophoricoside, found and isolated from the seeds of SJ, has shown promising activity against osteoporosis when tested in ovariectomized rat model [11,12]. Also a fruit extract, rich in isoflavones, was able to enhance the production of the growth factors TGF-β and IGF-1 in bone marrow cells from rat model [13]. 

Moreover, an extract of the flower buds was able to suppress the activation of microglia, the expression of IL-1β, and cell apoptosis. This led to decreased cerebral infarction area in a model of ischemia-reperfusion injury [14]. Furthermore, an ethanol flower bud extract was active against different bacteria like *Propionibacterium avidum*, *Propionibacterium acne*, and *Staphylococcus aureus* [15]. On the other hand, administration of mature fruits in a fatty diet to obese mice caused a decrease in body weight gain, high-density lipoprotein cholesterol, and total serum cholesterol levels [16].

Some flavonol triglycosides, which were isolated from fruits, have shown antioxidant effects in 2,2-diphenyl-1-picrylhydrazyl (DPPH^•^) and cytochrome-c assays [17]. However, few studies focused on the antioxidant properties of the plant. Therefore, this study aimed at further investigation of these properties.

In the present study, hydroalcoholic extract from the dried fruits of SJ was tested for its in vitro antioxidant activities using DPPH assay. The secondary metabolites of the bioactive extract were characterized using high performance liquid chromatography-photodiode array-electrospray ionization-mass/mass (HPLC-PDA-ESI-MS/MS). Furthermore, antioxidant activities were further investigated using two different in vivo models, *Caenorhabditis elegans* (*C. elegans*) and trimethyltin (TMT)-induced neurotoxicity model in mice.

*C. elegans* nematode is a vastly used successful model system for screening different agents regarding their antioxidant and neuroprotective activities [18,19,20]. Using this model, the ability of the extract to increase stress resistance and to modulate stress response genes expression, heat shock protein-16.2 (Hsp-16.2) and superoxide dismutase-3 (Sod-3), were investigated in our study. The levels of the antioxidant enzymes SOD, heme oxygenase-1 (HO-1), of glutathione (GSH) and of the oxidative stress marker malondialdehyde (MDA) were assessed using brains of TMT-stressed mice.

## 2. Results and Discussion

### 2.1. Chemical Characterization of SJ Extract

**Figure 1:**
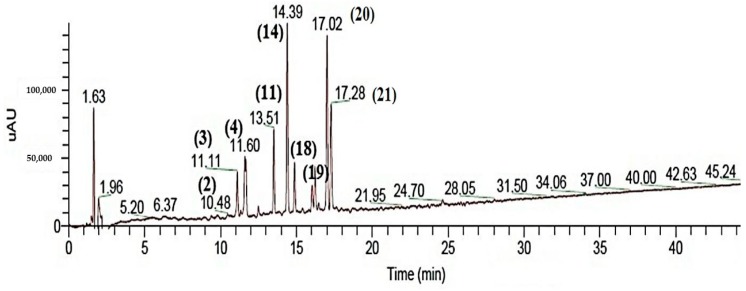
HPLC-PDA-ESI-MS/MS chromatogram for metabolites detected in a hydroalcoholic fruit extract of SJ (negative mode).

Phytochemical analysis of the hydroalcoholic fruit extract of *Styphnolobium japonicum* was performed using HPLC-PDA-ESI-MS/MS technique, see Figure 1 [21]. Several classes of secondary metabolites were detected as phenolic acids, flavones, flavan-3-ols, and tannins.

Several apigenin glycosides were represented in the bioactive extract. The major compound was represented in peak 14. Peak 14 was tentatively identified as apigenin 7-*O*-(2″-dihydrogalloyl)-deoxyhexosyl-6-C-(2″′-pentosyl)-hexoside (Table 1), as the deprotonated ion appeared at *m/z* 862. Its major ion fragment appeared at *m/z* 700, indicating loss of a hexose molecule [22].

Peak 1, [M − H]^−^ at *m/z* 639, was identified as apigenin acetyl dihexoside. Fragment ion peaks are present at *m/z* 431, which corresponds to apigenin hexoside and at *m/z* 638 corresponding to [M − 2H]^−^. Moreover, fragment ion peak at *m/z* 592 indicates apigenin dihexoside moiety and loss of acetyl group [M − H − 44]^−^.

Peak 10 was identified as apigenin hexoside, its deprotonated molecule appeared at *m/z* 431. The major fragment ion appeared at *m/z* 269 indicating apigenin aglycone [23]. Furthermore, peak 13 was recognized as apigenin 7-*O*-rutinoside (isorhoifolin). It is identified according to its deprotonated molecule at *m/z* 577 and its molecular ion peak of [M − H − 308]^−^ at *m/z* 269, due to rutinose loss [24].

Moreover, several kaempferol glycosides were recognized. Peak 4, representing one of the major compounds, was tentatively identified as kaempferol 3-*O*-[6″-*O*-(hexosyl) hexoside] 7-*O*-deoxyhexoside, as its [M − H]^−^ appeared at *m/z* 755. Several molecular ion peaks were shown at *m/z* 609, 431 and 284 representing loss of deoxyhexose [M – H − deoxyhex]^−^, two hexose moieties [M − H − hex − hex]^−^, and the presence of kaempferol moiety, respectively.

Kaempferol dihexoside was identified as peak 5, [M − H]^−^ at *m/z* 609. Two deprotonated fragments were shown at *m/z* 429 and *m/z* 284 representing losing one hexose [M − H − hex]^−^ and two hexose moieties [M − H − hex − hex]^−^, respectively.

Peak 2 was tentatively identified as kaempferol-3-*O*-rutinoside-7-*O*-*β*-D-hexopyranoside, [M − H]^−^ at *m/z* 755. That was confirmed via the observed fragmentation pattern as *m/z* 754 due to [M − 2H]^−^. In addition, *m/z* 609, *m/z* 593, and *m/z* 285 appeared representing the loss of deoxyhexose [M − H − deoxyhex]^−^, hexose [M − H − hex]^−^, and the presence of kaempferol moiety respectively [25].

Peak 8 was identified as kaempferol-feruloyl-di-hexose-pentose, [M − H]^−^ at *m/z* 931, and [M − H − 146]^−^ at *m/z* 785 indicating the loss of a deoxyhexose moiety [26].

The tentative identification of 24 compounds based on their retention values, MS data, and fragmentation pattern (MS daughter ions) is shown in Table 1.

### 2.2. Total Phenolic Content and Evaluation of Antioxidant Activity In Vitro

Total phenolic content was determined by Folin–Ciocalteu. The extract presented 192.4 ± 4.8 µg of gallic acid equivalents (GAE)/mg of SJ extract. This value was higher than that observed using aqueous extract of the plant and comparable to that obtained with 50% methanloic extract, using catechin standard [35].

Free radical scavenging activity of SJ extract was evaluated using DPPH^•^ assay, showing relatively high value (EC_50_ of 110.36 ± 3.18 µg/mL) compared to standard antioxidants; (−)-epigallocatechingallate (EGCG) (EC_50_ of 1.4 ± 0.06 µg/mL) and vitamin C (EC_50_ of 2.7 ± 0.09 µg/mL).

### 2.3. In Vivo Antioxidant Activity Using the C. elegans Model

#### 2.3.1. Survival Assay

**Figure 2:**
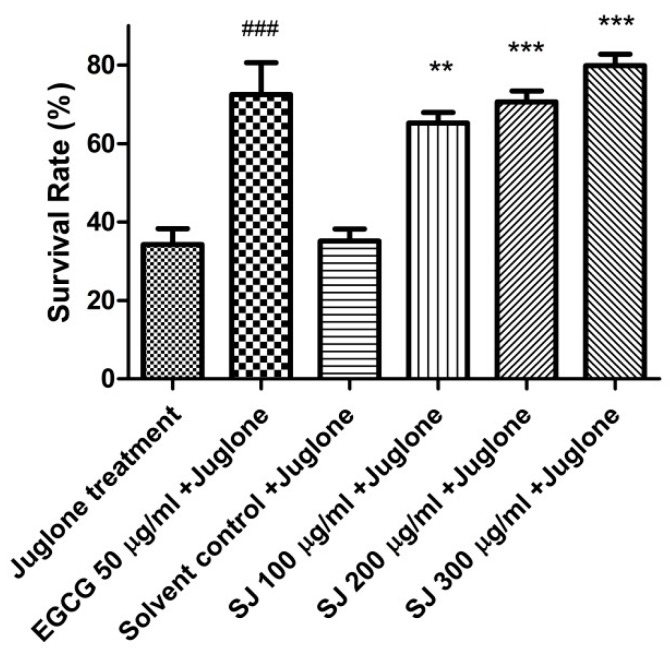
Effect of SJ extract on the survival rate of N2 worms pretreated with different doses of the extract and then subjected to oxidative stress via a toxic dose of juglone. SJ 300 µg/mL + juglone group showed a highly significant increase in rate of survival compared with solvent control + juglone group. Data is represented as mean ± SEM of survival rate % (*n* = 5) obtained by one-way analysis of variance test (ANOVA) followed by Bonferroni’s method of correction. ** *p* < 0.01 and *** *p* < 0.001 in comparison with solvent control + juglone group. ^###^ *p* < 0.001 in comparison with juglone group.

Several assays were employed to evaluate the antioxidant effects of SJ extract in vivo using the widely known *C. elegans* model. For the survival assay, a lethal dose of the naphthoquinone juglone was applied as a pro-oxidant for the induction of severe oxidative stress condition. Worms that were pretreated with SJ extract demonstrated significantly higher rates of survival, compared with the solvent control group. The highest dose of the extract—SJ 300 µg/mL—demonstrated the highest survival rate among all the tested doses, 79.86 ± 2.87%, compared to the juglone group (35.15 ± 3.06%, *p*-value < 0.001) (Figure 2). This result was even higher than that of the EGCG positive control group (72.46 ± 8.1%, *p*-value < 0.001). This data demonstrates the bioavailability of the extract.

Several flavonoids identified via HPLC-PDA-ESI-MS/MS, like kaempferol and proanthocyanidin derivatives, could be contributing to the observed effect. Kaempferol was reported previously to increase survival and resistance to thermal stress when tested in *C. elegans* [36]. Furthermore, plant extract rich in proanthocyanidins was able to enhance survival rates among the nematodes [37].

Moreover, increased survival rates could be due to the direct scavenge of reactive oxygen species (ROS) by the phenolic compounds of SJ extract or due to the activation of protective signaling pathways with subsequent expression of genes related to oxidative stress resistance. 

#### 2.3.2. ROS Intracellular Accumulation

To further assess the antioxidant potential of SJ extract in *C. elegans*, wild type (N2) worms were used for quantification of intracellular ROS levels. For the detection of ROS, the membrane permeable 2′,7′-dichlorofluorescein diacetate (H_2_DCF-DA) dye was employed. After deacetylation by cellular esterases, the dye is oxidized by ROS into 2’,7’-dichlorofluorescein (DCF), a fluorescent compound.

**Figure 3:**
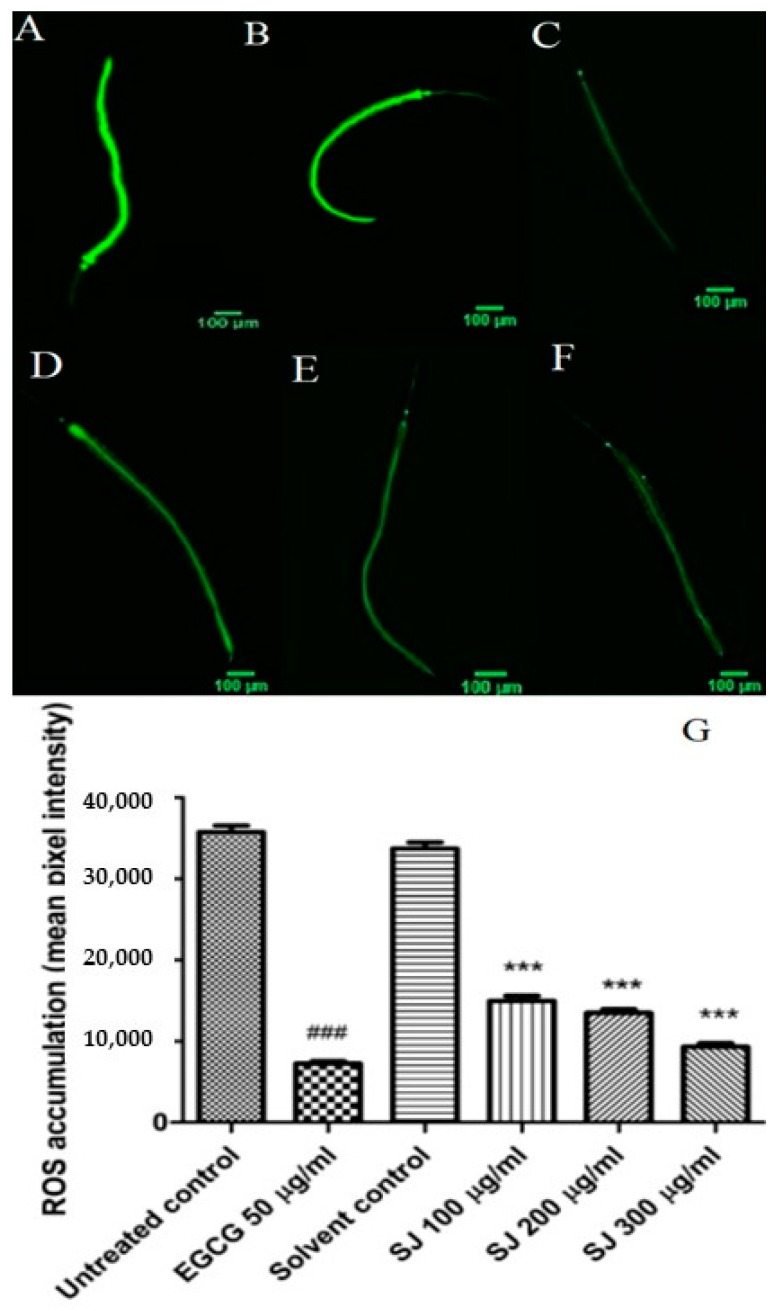
Intracellular reactive oxygen species (ROS) levels in *C. elegans*. (**A**–**F**) Pictures demonstrate intracellular ROS levels in worms: (**A**) untreated control, (**B**) solvent control, (**C**) (−)-epigallocatechingallate (EGCG) 50 µg/mL, (**D**) SJ 100 µg/mL, (**E**) SJ 200 µg/mL, (**F**) SJ 300 µg/mL, and (**G**) quantification of ROS levels using fluorescence intensity for different treated groups. SJ treatment resulted into lower ROS levels. Data represents the mean of three assays. Analysis was made through one-way ANOVA followed by Bonferroni’s method. *** *p* < 0.001 in comparison with solvent control and ^###^ *p* < 0.001 in comparison with untreated control.

Treatment with different doses of SJ extract resulted in a significant decline in ROS levels in comparison to solvent control group. SJ 300 µg/mL resulted in the highest decline in ROS levels, 72.3%, a result which is close to that of EGCG positive control group, 79.6% (*p*-value < 0.001) (Figure 3).

Proanthocyanidins, quercetin, and kaempferol derivatives, identified in the extract, could be participating in reducing ROS levels. Those flavonoids have previously reduced ROS accumulation in the *C. elegans* model [36,37,38].

As hypothesized for the survival assay, in addition to direct ROS scavenging, the activation of stress resistance related genes mediated by different compounds within the extract can also be involved in the observed diminution of ROS levels by SJ treatment.

#### 2.3.3. Expression of Hsp-16.2 Gene 

**Figure 4:**
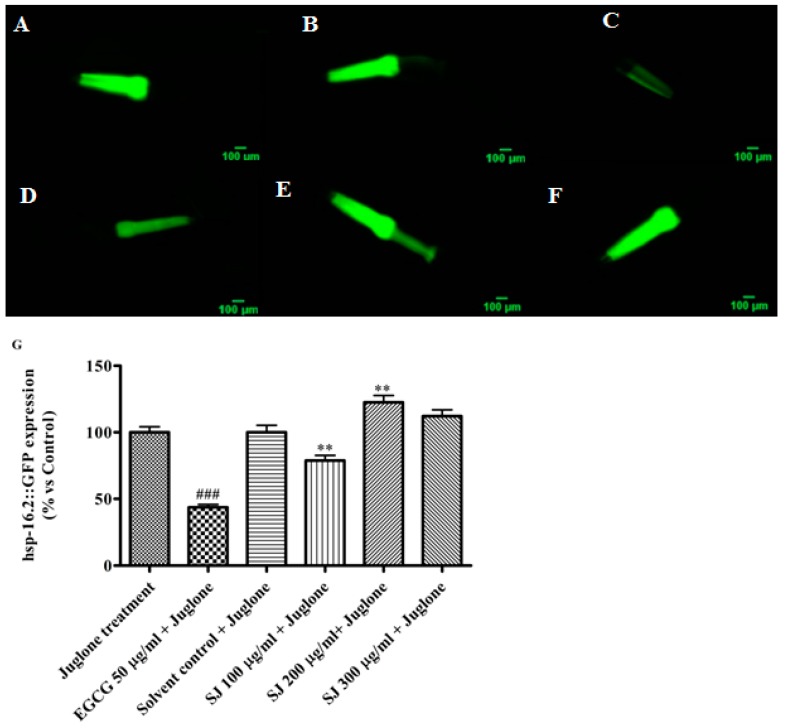
Hsp-16.2 gene expression in *C. elegans*. (**A**–**F**) GFP images of TJ375 worms from different treatment groups: (**A**) juglone group, (**B**) solvent control + juglone, (**C**) EGCG 50 µg/mL + juglone, (**D**) SJ 100 µg/mL + juglone, (**E**) SJ 200 µg/mL + juglone, (**F**) SJ 300 µg/mL + juglone, and (**G**) quantification of Hsp-16.2 levels using fluorescence intensity measurement for different groups. The lowest concentration of SJ extract used—SJ 100 µg/mL—resulted in significantly decreased expression of Hsp-16.2 gene. Data represent the mean of four assays. Analysis was performed through one-way ANOVA followed by Bonferroni’s method. ** *p* < 0.01 in comparison with solvent control + juglone and ^###^ *p* < 0.001 in comparison with juglone group.

The expression of Hsp-16.2, used as a marker of stress levels, was done using TJ375 mutants. These worms have Hsp-16.2 gene coupled to green fluorescent protein (GFP). This protein is inducible by applying different stressors, like heat and oxidative stress [39]. When nematodes are treated with juglone, a strong fluorescent signal becomes visible. Worms pretreated with the lowest dose of SJ extract, 100 µg/mL, then subjected to mild oxidative stress conditions using 20 µM juglone showed a very significant decrease in fluorescence intensity (21 ± 3.88%), in comparison to solvent control + juglone group (*p*-value < 0.01). EGCG group also showed a highly significant decline in fluorescence intensity (56.3 ± 2.08%) in comparison with juglone group (*p*-value < 0.001). On the other side, a higher SJ dose of 200 µg/mL lead to a significant rise in Hsp-16.2 levels (Figure 4).

Hormesis is defined as an adaptive process by which a low dose of a toxic compound leads to beneficial effects on the organism. Several antioxidants have demonstrated a hormetic dose response effect. They acted as pro-oxidants above certain doses, inducing the activation of antioxidant defense mechanisms and forming a state of stress resistance [40].

The elevated levels of Hsp-16.2, observed at concentrations above 200 μg/mL, might indicate a pro-oxidant activity of SJ extract causing a hormetic response. Comparable results were previously reported for several polyphenols that were able to trigger hormetic responses via modulating the expression of different Hsps [41].

In *C. elegans*, heat shock protein expression is regulated by the heat shock factor-1 (HSF-1) and DAF-16 transcription factor [42]. Therefore, further studies involving DAF-16 mutants will be needed for more understanding of the mechanism by which the extract exerts its effects.

#### 2.3.4. Expression of Sod-3 Gene

**Figure 5:**
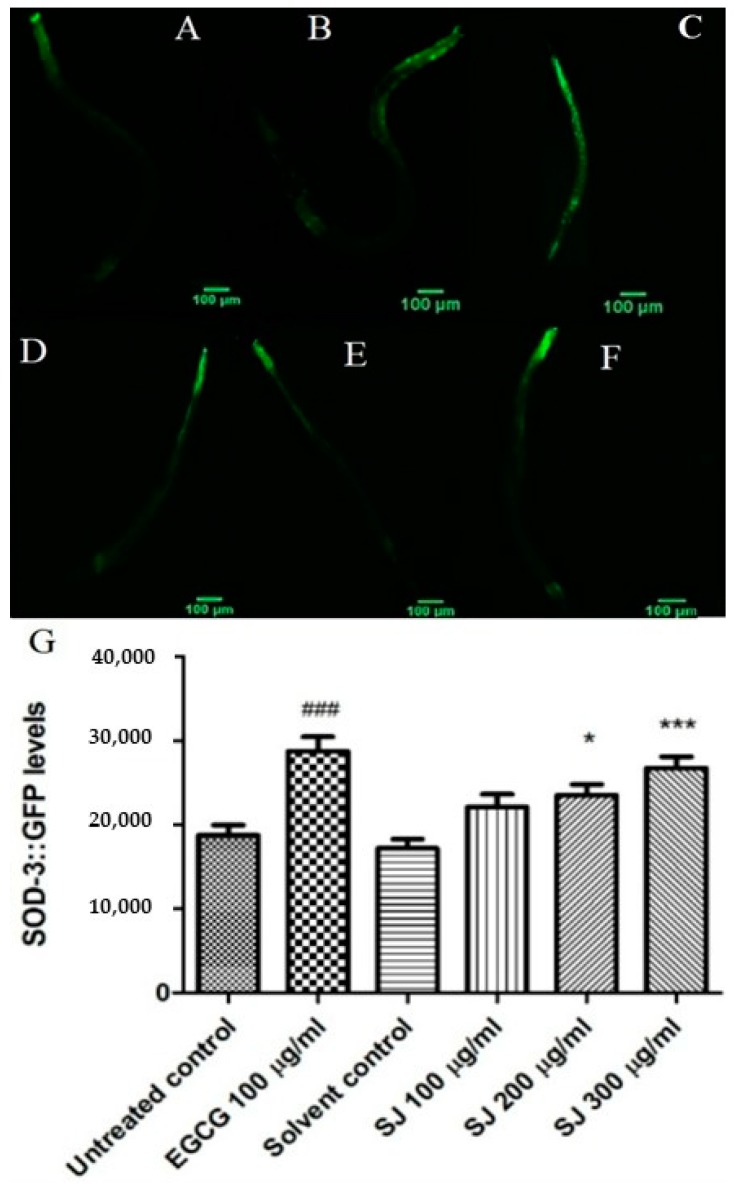
Expression of Sod-3 gene in *C. elegans*. (**A**–**F**) Photos of GFP fluorescence in the posterior part of the intestine of CF1553 worms from different treatment groups: (**A**) untreated control group, (**B**) solvent control, (**C**) EGCG 100 µg/mL, (**D**) SJ 100 µg/mL, (**E**) SJ 200 µg/mL, (**F**) SJ 300 µg/mL, and (**G**) quantification of sod-3 levels through fluorescence intensity measurement for different groups. The highest concentration of SJ extract used—SJ 300 µg/mL—resulted in significant expression of sod-3 gene. Data is represented as mean of three assays analyzed through one-way ANOVA followed by Bonferroni’s method. * *p* < 0.05, *** *p* < 0.001 in comparison with solvent control and ^###^ *p* < 0.001 in comparison with untreated control group.

SOD-3 enzyme expression was evaluated using the mutant CF1553 strain with sod-3 coupled to GFP reporter. Compounds with antioxidant activities often lead to elevation in sod-3 levels, which increases the superoxide anion radical (O_2_^-^) scavenging effect. SJ extract increased the levels of sod-3 in a dose-dependent fashion. The highest concentration tested of SJ extract, 300 µg/mL, shows the highest levels of SOD-3 enzyme (55.5%) in comparison with the solvent control group, whereas EGCG showed a 53.3% increase similar to that of SJ 300 µg/mL (*p*-value < 0.001) (Figure 5). Since sod-3 is directly regulated by DAF-16, the observed antioxidant effects might be due to the participation of DAF-16 transcription factor.

### 2.4. In Vivo Antioxidant Activity Using TMT Mouse Model

TMT is an organotin widely known for being used as a pesticide [43]. However, its neurotoxicity was revealed from previous reports of human intoxication with symptoms of amnesia, memory impairment and epilepsy [44,45,46]. Many studies reported its selectivity towards hippocampus. Therefore, it is considered a good model to be used for neuroscience research [43], especially in chronic neurodegenerative disorders like Alzheimer’s disease (AD) [47]. TMT neurotoxicity is mainly mediated via oxidative damage, resulting in the elevation of ROS and extracellular glutamate levels [48,49]. Moreover, a decrease in total SOD and GSH levels usually occurs after TMT intoxication [50,51]. Increasing ROS levels, as a result of TMT intoxication, is usually accompanied by lipid peroxidation with subsequent production of lipid peroxidation products like 4-hydroxynonenal and MDA [52]. Natural phytoconstituents, as vitamin C and quercetin, proved to exert neuroprotective activity via modulation of the oxidative damage induced by TMT [53,54].

In the current work, intraperitoneal injection of 2.5 mg/kg TMT in mice caused acute neurotoxicity within 24 h [55]. Neurotoxicity, possibly via oxidative damage, was demonstrated by the increased levels of the lipid peroxidation product MDA in the brains of mice in TMT group compared with saline control group. Moreover, the levels of the antioxidant enzymes SOD, HO-1 and of GSH were decreased significantly in TMT group in comparison with saline control. 

#### 2.4.1. Levels of the Oxidative Stress Marker MDA

MDA is a known toxic lipid peroxidation byproduct responsible for induction of mutagenicity through interaction with proteins and DNA [56]. It is considered as one of the commonly known biomarkers for detecting lipid peroxidation levels [57].

**Figure 6:**
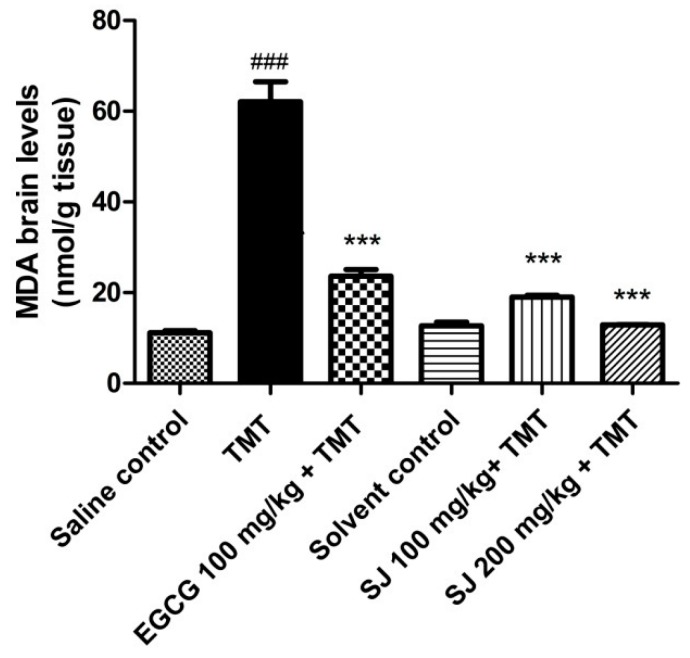
The effect of two different doses of SJ extract on MDA brain levels in mice intoxicated with TMT. The higher concentration of SJ extract used—SJ 200 mg/kg—resulted into higher decline in MDA levels. Statistical analysis was done via one-way ANOVA then Bonferroni’s method. *** *p* < 0.001 in comparison with TMT group and ^###^ *p* < 0.001 in comparison with saline control group.

Mice that received TMT manifested increment in the levels of MDA by 4.5-fold, compared to saline control group (*p* < 0.001). Mice pretreated with SJ extract showed significant depression in MDA levels as compared to the TMT group. The higher dose, SJ 200 mg/kg, showed a higher decline in MDA levels by 79.25 ± 0.09%; a result that is even higher than that of the group pretreated with EGCG (61.9 ± 1.47%, *p* < 0.001) (Figure 6). This result is similar to that observed in TMT mouse model using a *Ginkgo biloba* extract [58]. A proanthocyanidin-rich extract from *Cassia abbreviata* was also able to decrease MDA levels in rats [37]. 

The antioxidant effect exerted by SJ is probably due to the phenolic constituents found in the extract, like proanthocyanidins. They could be acting either directly through scavenging free radicals produced as a result of TMT injection, or indirectly via increasing antioxidant enzyme expression. A combination of both scenarios is also possible.

#### 2.4.2. Brain SOD Levels

SODs are a family of metalloenzymes that exert antioxidant effect via detoxifying O_2_^.-^ to O_2_ and hydrogen peroxide (H_2_O_2_) that is further reduced to H_2_O through glutathione peroxidase or catalase enzymes [59]. Antioxidant enzymes, like catalase and SOD, have a well-known role in protecting the body from high ROS levels via exerting detoxification mechanisms [60]. 

**Figure 7:**
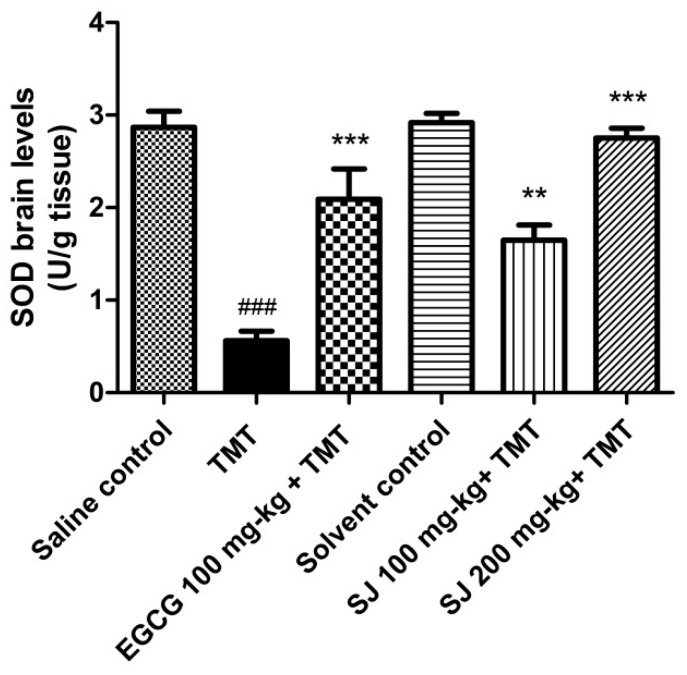
SOD enzyme levels in the brains of mice receiving two different doses of SJ extract then intoxicated via TMT. SJ 200 mg/kg treated group showed significantly higher SOD levels compared to TMT treated group. Statistical analysis was done via one-way ANOVA then Bonferroni’s method. *** *p* < 0.001 in comparison with TMT group and ^###^ *p* < 0.001 in comparison with saline control group.

To highlight on the possible involvement of antioxidant enzymes in the action of SJ extract, total SOD levels were measured in brains of mice. Acute administration of TMT to mice resulted into 80 ± 0.1% decline in the levels of SOD enzyme in the brains in comparison with saline control group (*p* < 0.001). This effect was reported previously using the TMT-induced neurotoxicity model [50,58]. Treatment with SJ extract counteracted the decline in SOD enzyme levels in a dose-dependent way. The used doses showed significantly higher SOD levels compared to TMT treated group. SJ 200 mg/kg group showed improvement in SOD levels by 92.9 ± 0.11% (*p* < 0.001). EGCG also demonstrated a significant elevation in SOD levels in comparison with that of TMT group (64.9 ± 0.32%, *p* < 0.001) (Figure 7).

Quercetin derivative could be contributing to the observed effect of the extract, as previous data showed the ability of quercetin to increase SOD levels in a mouse model of Parkinson’s disease [61]. 

#### 2.4.3. GSH Brain Levels

Glutathione is a well-known thiol containing nonenzymatic antioxidant compound found in the brain in its reduced and oxidized forms (GSH & GSSG) [62]. It protects the body from oxidative stress via direct scavenging activity of ROS and/or participation in some enzyme-catalyzed redox reactions to maintain redox homeostasis. Moreover, the absence of GSH in the brain increases the risk of oxidative stress and toxicity. Neurodegenerative disorders like AD, Parkinson’s disease (PD) and Huntington’s disease (HD) are characterized by imbalance in the levels of GSH in the brain. On the other side, low GSH levels is highly doubted to be one of the reasons for developing these diseases [63,64,65].

**Figure 8:**
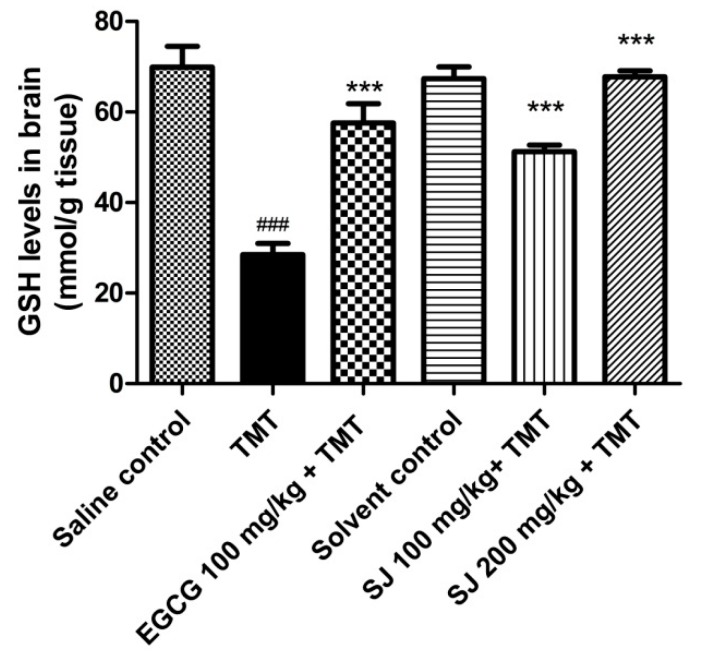
GSH brain levels in mice pretreated with SJ extract, at two different doses, then injected TMT. SJ 200 mg/kg group showed highly significant increase in GSH levels in comparison with TMT treated group. Statistical analysis was performed via one-way ANOVA then Bonferroni’s method. *** *p* < 0.001 in comparison with TMT group and ^###^ *p* < 0.001 in comparison with saline control group.

In the current study, the TMT treated mice demonstrated a highly significant decline of GSH to 59.16 ± 2.42% relative to saline control group (*p* < 0.001). Similarly, it was reported previously that the levels of GSH were reduced in the hippocampus of mice after administration of TMT [51]. However, groups treated with SJ extract showed higher GSH levels compared to TMT treated group. SJ 200 mg/kg treated group revealed 90.77 ± 1.32% elevation in GSH levels in the brain (*p* < 0.001). EGCG treated group showed highly significant result of 70.2 ± 4.24% elevation in GSH levels (*p* < 0.001) (Figure 8).

Previous administration of several herbal extracts resulted in elevating GSH levels, with a subsequent increase in the antioxidant potential and hepatoprotective activities in rat models of hepatotoxicity and neurodegenerative disorders like AD and PD [37,66,67].

#### 2.4.4. HO-1 Levels in Brain

HO-1 is an isoenzyme that belongs to the family of heme oxygenases. It is a stress-inducible antioxidant enzyme whose expression is induced via certain stressors like heavy metals, UV light, and heat shock. HO-1 activation is mediated via nuclear factor (erythroid-derived 2)-like 2/antioxidant response element (Nrf2/ARE) signaling pathway. It has a role in protecting neurons through catabolizing the pro-oxidant free heme to biliverdin, CO, and iron. This is essential to avoid the formation of free radicals that lead subsequently to cell death [68,69,70,71]. The increase in HO-1 expression is thought to increase oxidative stress resistance and to contribute in protection against cell death caused by oxidative stress [72]. 

**Figure 9:**
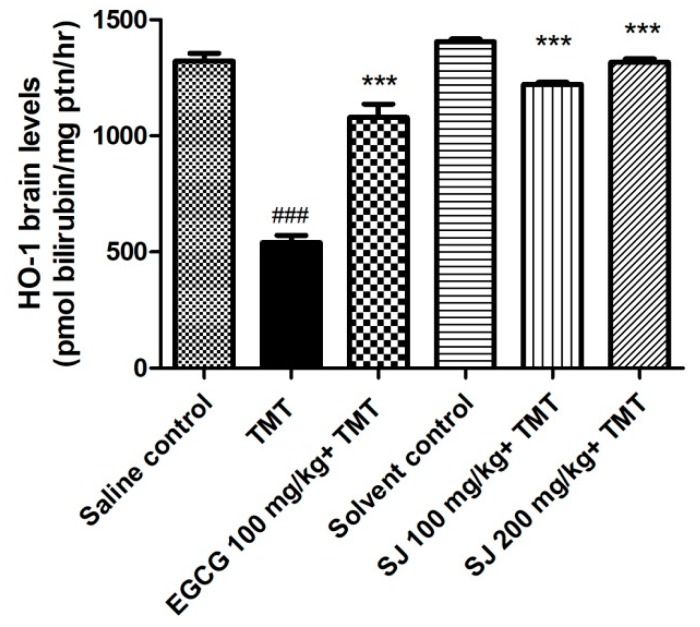
HO-1 levels in the brains of mice administering two different doses of SJ extract before being injected with TMT. SJ 200 mg/kg group demonstrated highly significant elevation in HO-1 levels compared to TMT treated group. Statistical analysis was done via one-way ANOVA then Bonferroni’s method. *** *p* < 0.001 relative to TMT treated group and ^###^ *p* < 0.001 relative to saline control group.

In this study, TMT injection caused a highly significant decline in HO-1 levels by 59% relative to saline control group (*p* < 0.001), which could be due to over consumption of the enzyme to detoxify ROS. Groups that received SJ extract showed high HO-1 levels compared to TMT treated group. SJ 200 mg/kg treated group demonstrated the highest increase in HO-1 levels (89%, *p* < 0.001). EGCG treated group also showed a highly significant increase in HO-1 levels (62.3%, *p* < 0.001) (Figure 9). The previous results highlight the possibility of the involvement of Nrf2/ARE pathway in the activity of the extract.

To sum up, the observed in vivo antioxidant activities using mouse model of neurotoxicity could be due to the variety of phenolic constituents present, like kaempferol and quercetin with their derivatives, which have shown neuroprotective properties in previous studies via decreasing MDA levels, increasing SOD, GPx, and HO-1 levels [73].

## 3. Materials and Methods

### 3.1. Plant Material

Dried ripe fruits of *Styphnolobium japonicum* were purchased from Kräuter Schulte, Germany. A voucher specimen of the plant, accession number 0047, is kept at Department of Pharmaceutical Biology in the Faculty of Pharmacy and Biotechnology at German University in Cairo.

The dried fruits were grounded, homogenized, and then extracted exhaustively with 70% methanol using agitated maceration at a temperature of 50–60 °C, for 24 h. Next, cotton wool was used for filtering the extract followed by two other rounds of extraction using the same conditions on the same plant material. The extracts were concentrated by being subjected to vacuum concentration using rotary evaporator at 37 °C. The product was freeze-dried, using a lyophilizer, and stored at −20 °C for further usage. 

### 3.2. Chemical Profiling of Phenolic Constituents with HPLC-PDA-ESI-MS/MS

Analysis of metabolites in SJ extract was done using HPLC-PDA-ESI-MS/MS. Thermo Finnigan LCQ-Duo ion trap mass spectrometer supported with an ESI source (Thermo Quest, Germany) directed to Finnigan Surveyor gradient HPLC system which compromises MS pump plus, autosampler (for automatic injection of the sample) and PDA detector plus was used. An EC 150/3 NUCLEODUR 100-3, reversed phase, C18ec column (Macherey-Nagel, Germany) was used for separation.

The mobile phase was composed of water and acetonitrile (ACN). The gradient employed was in the range of 5% to 60% ACN at 30 °C for 45 min, 0.5 mL/min flow rate. Sample volume of injection was 20 µL. MS detection was performed in the negative ion mode as previously described [18].

Full scan mode was applied with a mass range of 50 to 2000 *m*/*z*, using 35% collision energy for inducing fragmentation in MS/MS. Xcalibur software (Xcalibur^TM^ 2.0.7, Thermo Scientific, Waltham, MA, USA) was used for analysis and acquisition of data.

### 3.3. Antioxidant Activity In Vitro

#### 3.3.1. Measurement of Total Phenolic Content

Folin–Ciocalteu assay was used for evaluating total phenolic content of the extract using a 96-well microplate [74]. Twenty microliters of the extract or gallic acid standard were added. Afterwards, the addition of 100 µL of Folin–Ciocalteu reagent (Merck, Darmstadt, Germany) took place. After waiting at room temperature for duration of 5 min, 80 µL of 7.5% solution sodium carbonate were put to all wells. The reaction was left to take place at room temperature for duration of 1 h, away from light. Finally, absorbance measurement was done at 750 nm via microplate reader. The assay was done in duplicates, three times. Expression of phenolic content is done as gallic acid equivalents (μg of GAE/mg of SJ extract).

#### 3.3.2. DPPH^•^ Assay

This method was done to test for free radicals scavenging ability of SJ total extract in vitro. The assay was done using Blois method in a 96-well microplate [75]. One-hundred microliters of different concentrations of SJ, EGCG, and vitamin C were added followed by 100 μL of 200 μM DPPH^•^ solution (Sigma-Aldrich, Germany). Absorbance was measured at 517 nm after incubating the plate away from any source of light, for 30 min, at room temperature. A blank was prepared and measured for each sample in the same way, but without DPPH^•^ addition. The blank value was deducted from that of DPPH^•^. All measurements were done in duplicates and the assay was repeated three times. The ability to scavenge radicals was calculated via the following equation.

DPPH^•^ scavenging ability (%) = [(A_0_ - A_1_)/A_0_] × 100 (1)

A_0_ and A_1_ represent the absorbance of the control and the sample respectively.

EC_50_ was evaluated via sigmoid nonlinear regression in SigmaPlot and represented in µg/mL.

### 3.4. In Vivo Antioxidant Activity

#### 3.4.1. Strains and Culture Conditions of *Caenorhabditis Elegans*

All strains of *C. elegans* used in the current study and *E.coli* OP50 were bought from the Caenorhabditis Genetics Center (CGC), University of Minnesota, Minneapolis, MN, U.S.A.

All nematodes used in the study were cultured using nematode growth media (NGM) supplied with *E. coli* OP50 as their source of food and were preserved at 20 °C in an incubator. The used strains are as follows; wild type (N2), TJ375 (gpIs1[hsp-16.2::GFP]), and CF1553 (mu1s84[pAD76(sod-3::GFP)]). Age-synchronized worms were achieved as previously described [18,76].

#### 3.4.2. Survival Assay

Worms were sorted into six groups, 75–80 worms in each. First and second groups were used as negative controls. The first group was not treated: untreated control. This group is used for comparison with the solvent control group to ensure the absence of any activity resulting from the solvent used for dissolving the extract. The second group was the solvent control group and was treated with the same solvent used for dissolving the extract: 2.1% methanol. The third one was that of the positive control, EGCG 50 µg/mL. The last three groups were given different concentrations of SJ extract dissolved in 2.1% methanol: 100, 200, and 300 µg/mL respectively. All groups were incubated at 20 °C for 48 h. Afterwards, a toxic concentration of 80 µM juglone (Sigma-Aldrich, Germany) was added, a pro-oxidant used for oxidative stress induction. All worms were left for 24 h then dead and alive ones were scored. The dead worms are those which did not react to gentle repetitive stimuli with a platinum wire [77]. Survival assay was repeated five times and results were calculated and recorded as mean ± SEM of survival rate %. Analysis of results was performed with one-way ANOVA and Bonferroni’s method (post hoc).

#### 3.4.3. ROS Assay

Age-synchronized N2 worms at L1 stage were kept at 20 °C incubator in S-medium supplied with *E.coli* OP50. They were classified into six groups and treated as described before. Forty-eight hours later, all worms were washed using M9 buffer. After washing, 1 mL of 50 µM H_2_DCF-DA (Sigma-Aldrich, Germany), a dye used as an indication for ROS intracellular levels, was added to all groups. Afterwards, incubation was done at 20 °C for one hour in the absence of light. Finally, nematodes were washed using M9 buffer for the removal of excess dye. Fixing was done on glass slides using 10 mM sodium azide (AppliChem GmbH, Darmstadt, Germany) to induce paralysis. Fluorescence microscope (BIOREVO BZ-9000, Keyence Deutschland GmbH, Neu-Isenburg, Germany) supplied with mercury lamp at λ_ex_ 480/20 nm, λ_em_ 510/38, was used to take live images from the worms using constant time of exposure and 10X objective lens [19]. At least 30 worms were photographed from each group and the relative fluorescence intensity was determined for the full body of the worm densitometrically via Image J, version 1.48 (National Institutes of Health, Bethesda, MD, USA). Three different runs were made and results were presented as average fluorescence intensity. Results were compared via one-way analysis of variance test (ANOVA) followed by Bonferroni’s method of correction.

#### 3.4.4. Expression of Hsp-16.2::GFP

Transgenic age-synchronized TJ375 worms at L1 stage carrying GFP fused to Hsp-16.2 were used in this assay. Worms were sorted into six groups and treatment was performed as mentioned previously. All groups were left at 20 °C for 72 h. Afterwards, a sub-lethal juglone dose (20 µM) was added to all groups and worms were kept at 20 °C for another 24 h. 10 mM sodium azide was used for mounting the worms on glass slides [18]. Photos of 30 worms per group were taken using fixed time of exposure. Four different assays were performed and fluorescence intensity of the head was scored densitometrically via Image J. Results were analyzed and the mean fluorescence intensity was compared as previously mentioned.

#### 3.4.5. Expression of Sod-3::GFP

Age-synchronized CF1553 transgenic L1 worms, where GFP is fused to Sod-3 gene, were utilized in this assay. They were sorted into six groups and treatment was done as previously mentioned, except for EGCG positive control group where a dose of 100 µg/mL was used. Seventy-two hours later, they were analyzed using fluorescence microscopy and 10 mM sodium azide was used to paralyze worms to be mounted on glass slides [78]. Twenty to thirty worms were photographed per group via 10X objective lens using a constant time of exposure. Relative fluorescence intensity for the posterior part of the intestine was densitometrically measured via Image J. Results of three assays were analyzed and mean fluorescence intensity was compared as described previously.

#### 3.4.6. Animals

Adult Swiss albino male mice, 10–12 weeks old and weighing 25–30 g each, were used in the current study. All mice were bought from the animal colony of the National Institute of Research in Cairo, Egypt. They were housed for a minimum of one week in the animal house present in the Faculty of Pharmacy, Cairo University, for acclimatization before performing experiments. Animals were kept in plastic cages (6 mice/cage), supplied with wood shavings, and they were allowed to freely access rodent food and water. They were housed in both humidity and temperature-controlled room (20–25 °C; 50–60%) and exposed to 12 h light/dark cycle. Experiments involving animals were done after acquiring the approval of the Ethics Committee of the Faculty of Pharmacy in Cairo University (approval no. PT 2425). All treatments were done according to the guidelines of the National Institutes of Health Guide for Care and Use of Laboratory Animals. All considerations were done to make animal suffering as minimal as possible.

#### 3.4.7. Experimental Design

A single TMT dose of 2.5 mg/kg was used for induction of neuronal damage in mice [55]. Mice were weighed and sorted randomly into six groups, 6 mice each. All injections were done intraperitoneally.

Group I: Mice were injected saline three times (the first injection followed by the second, 1 h later, then a third injection taken 2 h from the previous one). This group acted as normal (saline) control. Group II: Mice were injected saline. After 1 h, they were injected TMT (2.5 mg/kg, i.p.), and then 2 h later they were reinjected with saline. This group served as TMT group. Group III was positive control group; mice were injected 100 mg/kg EGCG 1 h before and 2 h after TMT injection. Group IV was solvent control group; mice were injected 0.7% MeOH at three time intervals 0 h, 1 h, and 3 h, respectively. Groups V and VI were injected 100 and 200 mg/kg SJ extract respectively, 1 h before and 2 h after TMT injection. 2 h following the last injection, half of the mice were sacrificed and the other half was sacrificed after 24 h [55].

Mice were euthanized by cervical dislocation and the brains were immediately excised, rinsed with cold saline, to remove R.B.C remnants, and blotted between 2 filter papers. All brains were weighed and kept in −80 °C for the assay of different antioxidant and oxidative stress markers.

#### 3.4.8. Biochemical Analysis

##### Estimation of MDA Brain Levels

Estimation of lipid peroxides content in the brain, in response to injury mediated through TMT, was performed via assaying the most common lipid peroxidation product, MDA. MDA brain levels were evaluated in brain homogenates using the method described by Ohkawa et al. where brain homogenates were centrifuged (6000× *g* for 10 min at 4 °C). Afterwards, the supernatant was mixed with 0.67% thiobarbituric acid solution (TBA), 1% phosphoric acid and incubated in a water bath (95 °C for 1 h). After cooling, absorbance was measured at 532 nm [79].

##### Estimation of SOD Brain Levels

SODs total enzyme levels—cytosolic and mitochondrial—were determined in the brain homogenates using colorimetric test reagent kit (Biodiagnostics, Egypt) following the methods previously described by Nishikimi et al. [80]. Homogenates for measuring total SODs levels were centrifuged at 10,000× *g* for 15 min at 4 °C. Afterwards, a mixture of phosphate buffer, nitroblue tetrazolium (NBT), and NADH were added to the supernatant. Finally, phenazine methosulphate (PMS) was added to initiate the reaction and the increase in absorbance was recorded at 25 °C spectrophotometrically for 5 min at 560 nm.

##### Estimation of GSH Brain Levels

GSH brain levels were assayed using GSH kit of Biodiagnostic, Egypt. The method, according to Beutler and Kelly, depends on the reduction of 5,5’-dithiobis-(2-nitrobenzoic acid) (DTNB) by the SH group in glutathione (GSH) to produce an intense yellow compound. The reduced chromogen is directly proportional to GSH concentration and its absorbance can be measured colorimetrically at 405 nm [81]. 

##### Estimation of HO-1 Brain Levels

HO-1 isoenzyme activity in the brain tissue was evaluated following the method of Abraham et al. [82]. Bilirubin, the end product of heme degradation, was extracted using chloroform followed by determining its concentration spectrophotometrically.

### 3.5. Statistical Analysis

GraphPad Prism software version 5.01 (GraphPad Software Inc., San Diego, California, USA) was used for statistical analysis. Statistical significance between various groups was detected using one-way ANOVA followed by Bonferroni’s (post hoc) method. A probability level of p less than 0.05 was considered as statistically significant. SigmaPlot 11.0 (Systat Software GmbH Erkrath, Germany) was used to calculate EC_50_ value in DPPH^•^ assay.

## 4. Conclusions

Chemical profiling of a hydroalcoholic fruit extract of *Styphnolobium japonicum* by HPLC-PDA-ESI-MS/MS revealed the presence of a wide array of phenolic constituents, which could be responsible for the potential stress resistance properties shown via the in vivo used models.

In *C. elegans*, SJ extract exerted strong stress resistance properties via increasing survival rates, after exposure to oxidative stress, and decreasing intracellular ROS levels. Moreover, the antioxidant activity was demonstrated by the increased expression of sod-3, a DAF-16-specific target gene. Further studies are required to investigate the involvement of DAF-16/FOXO pathway in the activity of the extract. 

Testing the extract on a mouse model—a mammalian system—has further confirmed the antioxidant and stress resistance activities. Furthermore, high levels of HO-1 suggest the possible intervention of Nrf2/ARE pathway in the activity of the extract. 

In summary, the extract possesses promising activities and might be beneficial as a complementary therapy in the treatment of diseases involving oxidative stress. However, further investigations are needed to unveil the exact molecular mechanisms behind these activities. Additionally, its efficacy and safety on humans still needs exploration. 

## Figures and Tables

**Table 1:** Characterization of secondary metabolites in the hydroalcoholic extract of SJ fruits by HPLC-PDA-ESI-MS/MS.

Peak no.	Tentative Identification	R_t_ (min)	UV-Vis (λ max)	Peak Area (%)	[M − H]^−^	Fragment Ions (Ms/Ms)	Ref.
1	Apigenin acetyl dihexoside *	9.45	272, 334	0.3	639	638, 592, 431	Tentative
2	Kaempferol-3-*O*-rutinoside-7-*O*-*β*-D hexopyranoside *	10.48	290, 348	0.5	755	754, 609, 593, 285	[25]
3	Digalloyl-HHDP-Hexose *	11.11	240, 272	1.8	784	633, 631, 301	[27]
4	Kaempferol 3-*O*-[6″-*O*-(hexosyl) hexoside] 7-*O*-deoxyhexoside	11.60	290, 348	2.8	755	609, 431, 284	[28]
5	Kaempferol dihexoside	12.30	278, 349	0.7	609	429, 284	Tentative
6	Quercetin 7-*O*-hexoside-3-*O*-Rutinoside *	12.53	255, 354	0.25	771	463, 300	[24]
7	Trihydroxy-dimethoxy-flavone hexosyl-hexoside	12.65	271, 342	0.3	654	473	[29]
8	Kaempferol-feruloyl-di-hexose-pentose *	12.98	266, 333	0.18	931	785	[26]
9	Kaempferol 3-*O*-[6″-*O*-(hexosyl) hexoside] 7-*O*-deoxyhexoside	13.38	290, 348	0.15	755	284	[28]
10	Apigenin hexoside *	13.4	270, 338	0.19	431	269	[23]
11	Kaempferol rutinoside	13.51	254, 367	2.3	593	285	[24]
12	Kaempferol-3-*O*-hexose-*O*-caffeoyl-*O*deoxyhexoside *	13.62	254, 330	0.2	755	284	[30]
13	Apigenin 7-*O*-rutinoside *	14.19	268, 334	0.1	577	269	[24]
14	Apigenin 7-*O*-(2’’-dihydrogalloyl)-deoxyhexosyl-6-C-(2″′-pentosyl)-hexoside *	14.39	232, 336	3.5	862	700	[22]
15	Kaempferol derivative	14.5	278, 330	0.08	654	284	Tentative
16	Kaempferol di-*O*-deoxyhexoside	14.55	246, 344	0.07	577	431	[31]
17	Kaempferol-*O*-deoxyhexoside	14.94	245, 344	0.05	431	284	[31]
18	Galloyl-bis-HHDP hexose *	14.98	256	1.8	935	633	[32]
19	Coumaroyl quinic acid *	16.35	264,325	1.72	337	191	[33]
20	Naringenin-*O*-hexose-*O*-deoxyhexose	17.02	281	1.85	578	433, 271	[34]
21	Naringenin-*O*-hexose	17.28	281	0.82	432	271	[34]
22	Apigenin 7-*O*-(2″-dihydrogalloyl)-rhamonsyl-6-C-(2″′-pentosyl)-hexoside *	18.2	232, 336	0.9	863	269	[22]
23	Proanthocyanidin tetramer *	40.59	278	0.1	1121	849, 831	[32]
24	Apigenin 6-C-hexosyl 8-C- (6″-*O*-methoxygalloyl)- hexoside *	43.07	272, 340	0.4	758	269	[22]

* Secondary metabolites recognized for the first time from Styphnolobium japonicum.
